# Visualization, Data Extraction, and Multiparametric Analysis of 3D Pancreatic and Colorectal Cancer Cell Lines for High-Throughput Screening

**DOI:** 10.3390/biomedicines14010108

**Published:** 2026-01-06

**Authors:** Mikhail A. Trofimov, Ilya P. Bulatov, Velemir S. Lavrinenko, Vladimir E. Popov, Varvara S. Petrova, Anton S. Bukatin, Stanislav F. Tyazhelnikov

**Affiliations:** 1 JSC BIOCAD, Intracity Municipality the Settlement of Strelna, ul. Svyazi, d. 38, Str. 1, Pomeshch. 89, 198515 Saint Petersburg, Russia; 2 Nanotechnology Research and Education Centre, Alferov Saint Petersburg National Research Academic University of the Russian Academy of Sciences, ul. Khlopina 8/3, 194021 Saint Petersburg, Russia; antbuk.fiztek@gmail.com; 3 Institute for Analytical Instrumentation of the Russian Academy of Sciences, ul. Ivana Chernykh 31-33, 198095 Saint Petersburg, Russia

**Keywords:** high-throughput screening, 3D cell culture, image analysis, cancer spheroids, feature weighting algorithm, principal component analysis, drug screening, CellProfiler, Python

## Abstract

**Background:** Three-dimensional (3D) cancer models are currently essential tools in high-throughput screening (HTS), serving as a bridge between in vitro and in vivo approaches during drug development. Even though spheroids offer many advantages over 2D cultures, analyzing 3D cultures with heterogeneous morphology remains challenging due to the lack of standardized visualization techniques and multiparameter analysis. **Methods:** In this work, an optimized CellProfiler pipeline and a Python algorithm for weighting morphological features are used to visualize, extract, and analyze morphological data from spheroids derived from colorectal and pancreatic cancer cell lines with diverse morphologies (HCT116, LoVo, PANC-1, and CFPAC-1). **Results:** We developed a feature weighting process that combines multiple morphological parameters into a single metric using principal component analysis (PCA). There is a strong correlation between this process and a standard Alamar Blue proliferation assay (r = 0.89, ρ = 0.91, *p* < 0.001). Using this method, we were able to ascertain the IC_50_ values of substances that did not produce results in cell lines with heterogeneous morphology (LoVo and CFPAC-1) using a standard proliferation assay. **Conclusions:** By removing the need for tracer dyes, the resulting methodology may lower costs while accelerating preclinical drug development through informative multiparameter analysis of compound efficacy.

## 1. Introduction

Today, high-throughput screening (HTS) plays a major role in early drug development as a tool for rapid analysis of hundreds and thousands of compounds and selection of the most active ones, in particular on cells [1]. The development of HTS technologies has progressed due to improved automation processes, miniaturization of volumes, and increased speed and quality of data processing [2]. Currently, HTS mainly uses 2D cell cultures; however, their use limits the predictive power of the method due to the lack of intercellular interactions, concentration gradients, and other microenvironmental factors, and reduces the success of clinical trials to a maximum of 50%, according to various estimates [3,4,5,6]. For this reason, in recent years, there has been increasing interest in three-dimensional (3D) culture models, which provide more physiological conditions compared to 2D cultures. Three-dimensional cell models, which include spheroids and organoids, make it possible to create a more realistic microenvironment and functional characteristics, which makes it possible to obtain more high-quality information about the effect of a substance [7,8,9,10]. Despite a number of advantages, technical challenges persist, such as low compatibility with automated screening equipment, high reagent costs, and difficulties in data analysis. For these reasons, 3D cultures are still underrepresented in HTS [11,12,13,14,15].

Modern studies demonstrate that 3D cultures, when used in HTS, demonstrate significantly higher accuracy in predicting clinical trial outcomes compared to the standard process with 2D cells [16]. This correlation can be explained by the creation of physiological gradients of oxygen, nutrients, and therapeutic molecules within spheroids, which more closely match the tumor microenvironment [17]. However, the implementation of 3D models in industrial high-throughput screening systems is limited by technical difficulties in data collection and analysis: the signal reproducibility of standard screening protocols on spheroids is lower than that of 2D cultures, the process of obtaining uniform spheroids is more labor-intensive, and 3D cultures are more sensitive to mechanical stress [18]. New methods for extracting and analyzing multidimensional data from 3D cultures in a standardized and reproducible manner may provide a solution to these problems.

To improve the efficiency of 3D culture HTS, advanced methods for multiparametric image analysis are needed, requiring improved visualization techniques [19,20,21]. Fluorescence microscopy of 3D cultures offers the opportunity to collect data on cell geometry, viability, and other characteristics that are critical for assessing the efficacy of candidate compounds. However, existing data acquisition processes remain expensive, labor-intensive, and time-consuming due to a lack of new approaches [22,23,24,25]. Nevertheless, there are rapidly developing new methods for automated image analysis for the qualitative and quantitative assessment of cellular responses to candidate compounds, potentially accelerating drug development [26,27]. Recent advances in automation and image processing algorithms have significantly improved the analysis of 3D cultures [28]. For example, multiparametric phenotypic screening—in which cells are stained with multiple labels after exposure to a substance and phenotypic changes are assessed—allows for the simultaneous evaluation of the effects of compounds at the cellular and molecular levels. Such an approach allows not only to determine efficacy but also to suggest the mechanism of action of candidate cells [29]. However, it is rarely used in 3D cultures due to the high cost of materials and instruments, as well as the difficulty of interpreting the effects of 3D cultures compared to 2D cultures [20]. Therefore, the development of methods for analyzing high-throughput screening results, particularly for spheroids, remains relevant.

The open-source software CellProfiler is increasingly being used as a tool for extracting and analyzing optical and fluorescent images, enabling the evaluation of cell morphology, phenotypic characteristics, and structural changes [30,31,32]. Despite its potential, relatively few studies have focused on using CellProfiler for HTS analysis [33,34,35,36], especially in 3D culture-based screening [37,38], since the output contains a large number of parameters, the impact of which is difficult to individually assess and select the most significant ones, even for experienced specialists.

While some studies have examined individual morphological characteristics of spheroids as markers of response to candidate compounds [39], there are virtually no studies integrating multiple morphological parameters into a single metric. Principal component analysis (PCA) has been successfully used to solve various problems to reduce dimensionality while preserving data variance [40,41], and it offers the potential to reduce dimensionality and consolidate diverse morphological characteristics into a single metric with unbiased assignment of coefficients [42].

While multiparametric, image-based profiling pipelines, in particular those based on CellProfiler and Cell Painting–type high content assays, are by now well established in biomedical image analysis, they predominantly rely on multichannel fluorescence imaging and are optimized for 2D cultures [31,34,36,43] or homogeneous 3D systems [37,44]. In contrast, the workflow proposed in a current study is specifically designed for heterogeneous 3D spheroids and relies on label-free brightfield images combined with a PCA-weighted composite metric. Unlike classical dimensionality reduction approaches, primarily used for visualization or clustering, PCA is used here to derive feature weights for a unified response metric, with a conditional inversion strategy to accommodate features with opposing response trends. Importantly, this allows quantitative assessment of compound effects in spheroid models where conventional viability-based IC_50_ estimation is unreliable or fails, as illustrated for LoVo and CFPAC-1 cell lines. The combination of these elements differentiates the present pipeline from previously reported 3D multiparametric image-based screening strategies [37,44].

Thus, the development of visualization and multiparametric analysis of spheroid data exposed to candidate compounds may provide a useful tool for high-throughput screening. The aim of this study is to develop a method for visualizing three-dimensional cancer cultures of various morphologies using a visualization system with subsequent capabilities for high-throughput drug screening.

## 2. Materials and Methods

### 2.1. Cell Lines and Culture Conditions

Colorectal cancer cell lines HCT116 and LoVo, pancreatic cancer cell lines PANC-1 and CFPAC-1, were purchased from the American Type Culture Collection (ATCC) and cultivated according to the manufacturer’s instructions in DMEM nutrient medium (Gibco, 12-100-061, Grand Island, NY, USA) supplemented with 10% Heat Inactivated Fetal Bovine Serum (FBS) (Thermo (Gibco), 16140-071, Grand Island, NY, USA) and 2 mM of L-glutamine (PanEco, F032, Moscow, Russia). For all experiments, cell lines not later than the 20th passage were used.

### 2.2. Data Collection for Spheroid Analysis Under Cytostatic Exposure

To prepare 3D spheroids, cells were prepared at a concentration of either 100, 500, or 1000 cells per well, depending on the cell line (Table 1) [37,45,46,47,48]. The resulting suspension with a total volume of 75 μL was then transferred to a 3D cell 96-well ultra-low attachment round-bottom culture plate (SPL Lifesciences, cat. No 34896, Naechon-Myeon, Pocheon-si, Gyeonggi-do, Republic of Korea) by slow centrifugation at a rate of 400 rpm for 5 min. Then the cells were left to grow for 72 h at 37 °C in a humid atmosphere with 5% CO_2_. All liquid handling steps were performed manually; however, the protocol was designed to be compatible with automated liquid handling systems for future scale-up.

For further analysis of the selected cell cultures and optimization of screening conditions in CellProfiler, we investigated the effects of well-known anticancer drugs (paclitaxel, 5-fluorouracil, cytarabine, niraparib, etoposide, and oxaliplatin) synthesized in the BIOCAD laboratory, with the exception of 5-fluorouracil (5-FU) (Sigma-Aldrich, F6627, St. Louis, MO, USA) and etoposide (Abcam, ab120227, Waltham, MA, USA). All compounds were stored as 10 mM stock in DMSO before use.

On the third day of spheroid incubation, titrations of pre-prepared active substances in 75 μL (1:1 ratio with volume in wells) of fresh cell medium were added to wells at concentrations ranging from 0–10 μM (0–1 μM for paclitaxel) (ten points). After 21 days of incubation, fluorometric data were collected following incubation with the proliferation reagent Alamar Blue (ThermoFisher Scientific, DAL1100, Carlsbad, CA, USA) added according to the manufacturer’s instructions. Incubation time of spheroids with Alamar Blue was from 6 to 20 h, based on the specific requirements of each cell line. The fluorescence levels were assessed using an EnSight (PerkinElmer, Waltham, MA, USA) plate reader. On the same day, imaging data were obtained using the Celena X High-Content Imaging system (Logos Biosystems, Gyeonggi-do, Republic of Korea) with Celena X Explorer 1.6.0, followed by morphological parameter extraction of spheroids in CellProfiler 4.2.6.

Brightfield images were acquired using fixed acquisition settings within each experiment. Illumination intensity, exposure time, and 4× magnification were kept constant across all wells of a plate and across replicate experiments to minimize technical variability. No adaptive exposure or contrast enhancement was applied during image acquisition.

Given the label-free brightfield modality and the subsequent normalization and scaling of morphometric features prior to analysis, minor variations in absolute illumination levels were not expected to substantially affect the extracted morphological parameters.

All experiments were performed in at least three independent biological replicates (n = 3), with three technical replicates per condition. Experimental variability was maintained with coefficient of variation (CV) values below 20% across independent experiments for fluorescence units only [42,49,50,51,52].

### 2.3. Data Analysis

Images obtained with the Celena X High Content Imaging system with Celena X Explorer were processed in CellProfiler 4.2.6 for spheroid features extraction. A Python script was developed to consolidate CellProfiler-extracted features into one comparative metric. The developed script performed outlier detection, normalization, PCA with assigned weights to features, and calculation of a weighted comparative metric (Figure S1). This script was executed in Jupyter Notebook version 7.2.2 using Python 3.10. Calculated values of the extracted comparative metrics were analyzed using GraphPad Prism 9.5.1 software, creating dose–response curves and calculating IC_50_ values for each compound. The entire process, therefore, occurred within a modular, semi-automated process, which could be described in terms of distinct steps in several software applications. However, these steps are script-based and can be easily modified to run them batched or integrated into a fully automated procedure.

The CellProfiler process of feature extraction from images was carried out on an individual consumer computer (Apple MacBook Air; Apple M2 processor; 8 GB of RAM, Designed by Apple in Cupertino, CA, USA, assembled in China). The rate of image analysis on this computer was about 180 to 360 images per hour, depending on the morphological characteristics of spheroids in terms of complexity. The following statistical processing in either computer software (Python or GraphPad Prism) involved very little computing time and, therefore, was not a bottleneck for the pipeline.

For morphometric data, the outlier detection was performed using Dixon’s Q-test because this test is more robust when sample sizes are small (n = 3 for technical replicates with three biological replicates) and less sensitive to variations in data scale compared to coefficient of variation methods [53,54]. The Q-test was applied for computational data outlier detection according to the following formula:

Q = |suspect value − nearest value|/|highest value − lowest value|,

The calculated Q values were compared against critical Q values at a 95% confidence level.

To minimize the risk of excessive data exclusion inherent in outlier tests in small samples, the Dixon Q test was restricted to replacing one outlier value with the mean of the remaining values from each triplicate. This was applied only if the remaining values maintained the expected monotonic dose–response relationship.

This statistical approach provided a more objective criterion for outlier identification across different morphometric parameters with varying scales and distributions [53].

IC_50_ values were estimated by nonlinear regression using a four-parameter logistic (4PL) model implemented in GraphPad Prism. Data are presented as mean ± standard deviation (SD). Dose–response curves and IC_50_ values were fitted using nonlinear regression (GraphPad Prism 10.4.1). The relationships between the IC_50_ of the proliferation assay, the area, and the new metric were assessed for compounds for which IC_50_ could be determined, using Pearson’s correlation coefficient (r). In addition to Pearson correlation analysis, Spearman’s rank correlation coefficient (ρ) was used to assess monotonic associations between IC_50_ values derived from image-based metrics and those obtained from proliferation assays. To evaluate robustness, PCA-based feature weighting was recalculated using alternative cumulative explained variance thresholds (80% and 95%), and the resulting IC_50_ values were compared by correlation analysis.

Outlier handling was performed in an assay-specific manner, reflecting the fundamentally different nature of proliferation and image-based morphometric data. For the proliferation assay, the coefficient of variation (CV) was used exclusively as a quality-control criterion to identify unstable fluorescence measurements in triplicates, in accordance with established recommendations for resazurin-based screening assays [49,52]. In contrast, Dixon’s Q-test was applied only to morphometric features extracted from image analysis, where three technical replicates were available, and isolated segmentation or imaging artifacts could occur.

Importantly, CV- and Q-test-based procedures were not combined within a single decision step and were not applied to the same data. Each method was used independently within its respective analytical pipeline, and, therefore, no conflict resolution between the two approaches was required.

### 2.4. Mathematical Framework for Multiparametric Analysis

In order to overcome the disadvantages of a single-parameter analysis, we developed a mathematical framework integrating multiple morphological parameters. The process was divided into four major steps:

1. Normalization of extracted features to the [0, 1] range on control values:

x_norm = x/x_average_control × 100%

2. Feature transformation by conditional 1/x inversion for parameters that show reverse trends:

x_trans = 1/x_norm if mean (x_norm) > 1 and mean (1/x_norm) < mean (x_norm)

3. PCA for feature weighting: Components were selected to explain ≥90% of variance. The weight for each feature j was computed as follows:

ω_final(j) = Σ(a_i × |c_ij|),

Here, a_i is the explained variance ratio for component i, and c_ij is the coefficient of feature j for component i.

4. Computation of the final weighted metric:

σ_final = Σ(ω_final(j) × j)

The performance of the algorithm was evaluated by calculating Pearson correlation coefficients between the predicted values and reference proliferation assay results, and statistical significance was considered at *p* < 0.001.

## 3. Results and Discussion

### 3.1. Proliferation Analysis of Spheroids

**Figure 1:**
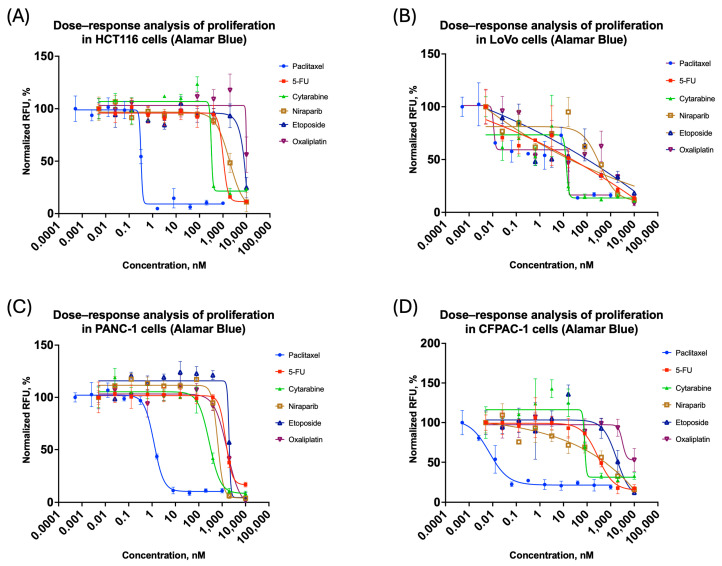
Three-dimensional cell cultures: antiproliferation curves on cytostatic response: (**A**) HCT116; (**B**) LoVo; (**C**) PANC-1; (**D**) CFPAC-1. All the curves represent the data as mean ± SD of at least three independent biological replicates (n = 3) with three technical replicates. The *x*-axis shows compound concentration (nM, logarithmic scale), and the *y*-axis represents normalized Alamar Blue relative fluorescence values (RFU).

To obtain comparative data on the proliferation properties of spheroids, we investigated the effects of widely used cytostatics with well-characterized action mechanisms: paclitaxel, cytarabine, 5-fluorouracil, niraparib, etoposide, and oxaliplatin. For the LoVo cell line, it was impossible to generate four-parameter inhibition curves for some cytostatics, probably because of high variability in the data. Figure 1 presents the proliferation inhibition curves obtained from these experiments.

Outlier detection using the coefficient of variation [54] was performed in order to analyze data before normalization. Deviation was calculated in MS Excel using the following formula:

Coefficient of Variation = (Std. Dev. of Triplicate)/(Mean Signal Val. of Triplicate) × 100%

Moreover, in the case of the LoVo cell line, at some concentrations, significant variation of more than 20% was recorded in triplicate measurements. Since this value was set as the criterion for the outlier, it followed that the data concerning this cell line was less reproducible to assess the proliferation effect as compared to others. The described effect might be a consequence of the irregular or non-spherical shape of the spheroids formed [42].

The following steps were performed to process the data from the proliferation assay:

1. Outlier identification from raw data:
  - If, after removal of one value, the standard deviation in a triplicate exceeded 20%, the point was excluded from analysis [55].
  - If an outlier was present within the IC50 range or the inflection point of the logarithmic curve, the graph was considered invalid [56].
  - A single exclusion was allowed while preserving the inhibition profile, provided that the outlier was located within the plateau region.
  - If the standard deviation in the triplicate of the negative control (no compound added) was greater than 20% and could not be normalized by removing a single point, then the plate corresponding to this control was considered invalid [56,57,58].
  - Outlier reproducibility and data loss evaluation: If outliers were systemic and resulted in repeated failures of plates or inconsistency in graphs, the cell line was considered “problematic”. In such cases, a large number of outliers can increase the CV to more than 20%, and the plate values will fail the acceptable threshold without total data loss [42,49].
2. In fact, due to the data collected, the LoVo cell line was highlighted as problematic. Figure 1B shows that after preprocessing, the coefficient of variation was very high, with outliers reaching over 20%. For such cases, the refined analysis developed in the current paper can be of special value.

**Figure 2:**
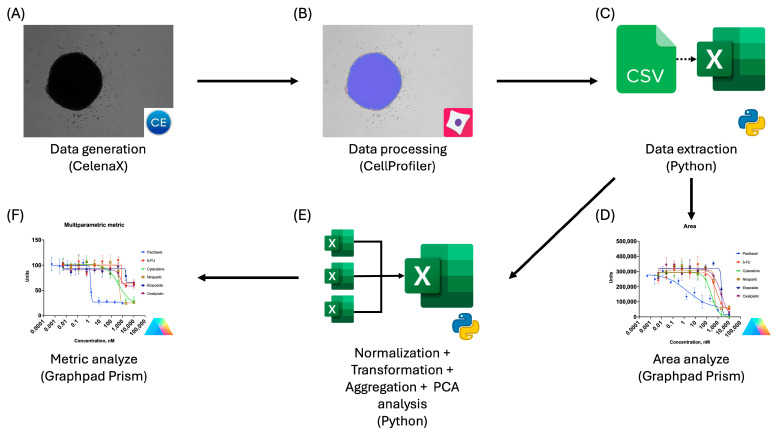
Workflow for preparation, data extraction, and analysis using CellProfiler: (**A**) Brightfield image acquisition using the Celena X imaging system; (**B**) extraction of spheroid morphological features using CellProfiler; (**C**) export of extracted features to tabular format (CSV/Excel) for downstream analysis using Python; (**D**) dose–response analysis of spheroid area changes induced by cytostatic treatment performed in GraphPad Prism; (**E**) multiparametric data processing, including normalization, transformation, aggregation, and principal component analysis (PCA), implemented in Python; (**F**) dose–response analysis of the derived multiparametric metric in GraphPad Prism.

Thereafter, the titrated cytostatic-treated plates were used for the optimization of optical analysis conditions in Celena X Explorer and extraction of data in CellProfiler, establishing a standardized pipeline of analysis. An overview of the workflow is presented in Figure 2. In order to obtain an image of the whole spheroid in one frame, imaging in Celena X Explorer was performed using a 4× objective while maintaining exposure settings specific to each cell line.

### 3.2. Optimization of Optical Analysis Conditions and Data Extraction in CellProfiler

For consistent image processing, while efficiently using available computational resources, a pipeline was adapted from the work of Alsehli et al. [38]. Acquired images were processed in CellProfiler; data were extracted for curve fitting according to this pipeline, whose module descriptions are presented in Table S1.

The original Alsehli et al. [38] pipeline included Expand or Shrink Objects, Split or Merge Objects, and Fill Objects modules for merging identified objects into spheroids; however, due to the use of an objective with lower magnification and the study of larger objects, accurate spheroid identification was achieved without the necessity for these modules. As a result, excluding these modules reduced segmentation artifacts and prevented occasional software crashes caused by excessively small objects or extensive void filling.

Morphometric criteria extracted for analysis included the following:

- Area Shape;
- Compactness;
- Form Factor;
- Median Radius;
- Perimeter;
- Solidity;
- Granularity–iterations, in which the signal change exceeded 5%.

A relative 5% threshold was applied to discard non-informative scales that correspond to an image background and minor brightness fluctuations. This number is empirical and was chosen as a practical threshold for the differentiation between meaningful texture signal and noise, based on recommendations given by the CellProfiler community and common practices in multiscale texture analysis [30,38,59,60]. Alternative threshold values were not systematically optimized for individual cell lines, as the goal was to apply a uniform and robust criterion across heterogeneous spheroid models. In preliminary inspections, substantially lower cutoffs increased sensitivity to segmentation noise, whereas higher cutoffs risked attenuating subtle but reproducible treatment-induced changes. Therefore, a 5% threshold was chosen as a pragmatic compromise balancing noise suppression and signal retention.

We consider spectral components with a <5% contribution to the maximum granularity amplitude as negligible and exclude them from further feature weighting. This criterion ensures that subsequent analysis focuses on structural details relevant to spheroid morphology while minimizing the influence of background irregularities.

To ensure correct image analysis and downstream processing, consistent file naming and metadata encoding are essential. Metadata (cell line, compound, concentration, and replicate identifiers) were automatically extracted from image file names using a regular-expression-based parser implemented in Python. The full regular expression and parsing logic are provided in Supplementary Materials (Table S2). For testing and refining data extraction formulae, the website pythex.org was useful, allowing the validation of extraction requests as well as the review of available functions for metadata processing [61].

**Figure 3:**
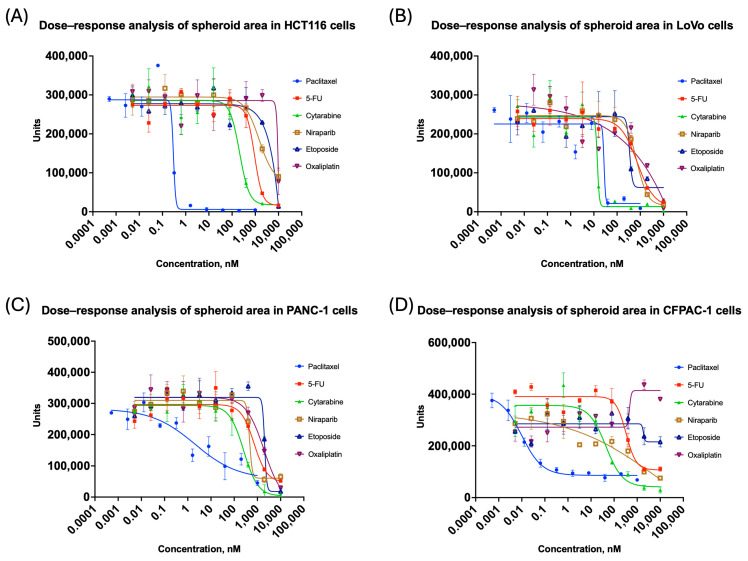
Changes in spheroid area for colorectal and pancreatic cancer cell lines grown in 3D: (**A**) HCT116; (**B**) LoVo; (**C**) PANC-1; (**D**) CFPAC-1. All the curves represent data as mean ± SD of at least three independent biological replicates (n = 3) with three technical replicates. The *x*-axis shows compound concentration (nM, logarithmic scale), and the *y*-axis represents area values. A decrease in area indicates spheroid shrinkage or fragmentation.

After outlier assessment using the coefficient of variation, graphs of spheroid area changes were generated (Figure 3), but for a more general approach, the contribution of all the features extracted had to be considered.

### 3.3. Multiparametric Analysis of Morphological Features of Spheroids Using a New Metric

**Figure 4:**
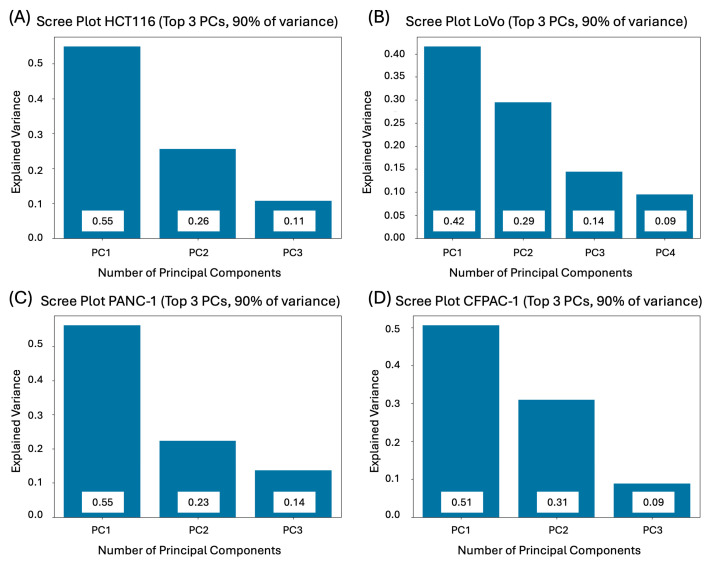
Required number of principal components to explain 90% of the variance in colorectal and pancreatic cancer cell lines cultivated in 3D: (**A**) HCT116; (**B**) LoVo; (**C**) PANC-1; (**D**) CFPAC-1.

**Figure 5:**
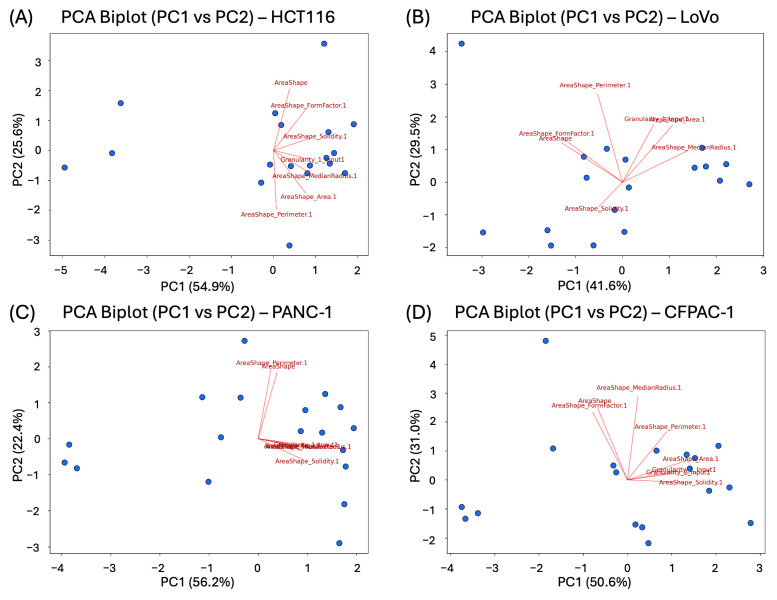
PCA Biplots of 1st and 2nd components in colorectal and pancreatic cancer cell lines cultivated in 3D: (**A**) HCT116; (**B**) LoVo; (**C**) PANC-1; (**D**) CFPAC-1. The first two principal components (PC1 and PC2) are shown, with axis labels indicating the percentage of total variance explained. Arrows represent individual morphological features; their direction reflects the sign of the feature loading, and their length indicates the relative contribution of the feature to the corresponding principal components. Points correspond to individual experimental conditions and concentrations, illustrating how spheroid phenotypes are distributed in the reduced component space.

A Python script was developed to compile the extracted features, which conducts the following:

- Extract all data from the final CSV file into an Excel-friendly format, showing values in positions corresponding to the well plate layout;
- Check all studied metrics for outliers using the Q-test (Dixon’s test) in triplicate. Since the coefficient of variation is sensitive to data scale and metrics can be very different in range, such as area shape within hundreds of thousands, while form factor is in single digits, certain adjustments had to be made accordingly [53,54];
- Normalize obtained features to a unified scale.
- Verify and transform features using the inversion x^−1^ method. If a feature’s mean value in a triplicate exceeded 1 after normalization, it indicated an increase in titration relative to the control. Since different features could trend differently with concentration, direct aggregation without transformation could misrepresent compound effects [43,62,63].
- Re-check the transformed features and reverse them if needed. In cases where the mean feature value rose after transformation, this signaled that either the original data remained constant or had just slightly decreased and should return to its initial value.
- Perform PCA to find the weights of the studied metrics. Giving feature weights manually would be problematic, as well as subjective, since different cell lines have different important features. The PCA enabled us to reduce the dimensionality and compute weight coefficients based on the explained variance, which is illustrated in Figure 4 and Figure 5 [40]. As there is no clearly accepted criterion for selecting the exact number of principal components in analysis [64,65], we adopted a cumulative explained variance threshold of 90%, following commonly used approaches in multiparametric and image-based PCA studies [66,67,68,69]. Feature weights were computed based on this.
- Finally, the script developed a comparative value per well by summing weighted feature scores, normalizing the total sum to 1, and generating the final weighted values corresponding to each metric.

One of the important steps in the computational analysis involved metric inversion to capture the growing trends at higher compound concentrations. If such an inversion were not performed, the final metric might fail to reflect expected trends. Some morphological features exhibit monotonic responses to compound treatment in opposite directions (e.g., increased form factor versus decreased spheroid area). To ensure consistent interpretation within a unified multiparametric metric, features whose response direction was inversely related to treatment-induced spheroid impairment were inverted (1/x) prior to normalization [43,63]. This procedure was applied based on the sign of the monotonic association between feature values and compound concentration and served solely to align feature directionality, not to alter their relative contribution in PCA.

Although PCA has been employed in various contexts [34,35,43,70,71], the method proved to be highly valuable in the context of automated and unbiased assignment of weights, data dimensionality reduction, and transition to a unified weighted metric.

**Figure 6:**
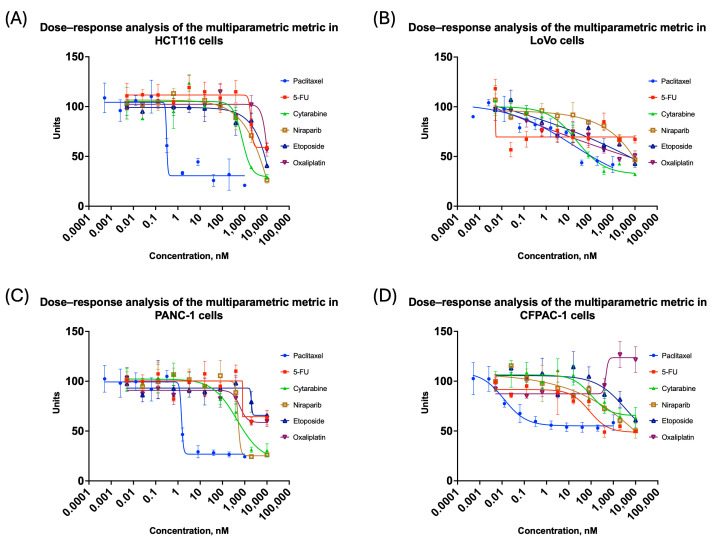
Changes in total weighted metric for colorectal and pancreatic cancer cell lines cultivated in 3D: (**A**) HCT116; (**B**) LoVo; (**C**) PANC-1; (**D**) CFPAC-1. All the curves represent the data as mean ± SD from at least three independent biological replicates (n = 3) with three technical replicates. Dose–response curves based on the integrated multiparametric metric. The *x*-axis shows compound concentration (nM, logarithmic scale), and the *y*-axis represents the normalized metric. A decrease in the integrated metric reflects increasing treatment-induced spheroid impairment.

The weighted values obtained for normalization were summed to obtain a single metric that characterized the changes in spheroid characteristics in compound-treated wells when compared to control wells. Figure 6 presents the results of spheroid image analysis and morphometric criteria for all studied cell lines.

The IC_50_ values for compounds tested in this study are collected in Table 2. It may be noted that in the case of the LoVo cell line, calculation of the IC_50_ value, based on proliferation activity, was not always possible because of a high variability. Determination of the moment of spheroid changes was more accurate when the spheroid area or the weighted comparative metric was used. Moreover, in the case of compounds that exerted relatively weak effects within the tested concentration range (e.g., etoposide or oxaliplatin in the HCT116 cell line), changes were detected earlier and more distinctly by morphological metrics than by measurement of proliferation activity.

As summarized in Table 2, IC_50_ values derived from the multiparametric morphological metric do not always coincide with those obtained from proliferation-based assays. These discrepancies might reflect differences in the biological processes probed by the two readouts. In fact, morphological responses of 3D spheroids—such as changes in size, compactness, surface irregularity, or internal structure—may precede detectable alterations in metabolic activity, particularly in the case of cytostatic compounds that induce growth arrest or structural remodeling without immediate loss of viability [72] as it was shown in case of 5-FU effect on pancreatic cancer cell lines with IC_50_ relation as >0.29 for PANC-1 and 0.22 for CFPAC-1. Furthermore, fluorometric proliferation assays in 3D spheroids may be affected by limited dye penetration, diffusion gradients, and metabolic heterogeneity within large or irregular aggregates, which can attenuate or delay the apparent drug response [73].

On the other hand, in some conditions, metabolic inhibition can be detected earlier than pronounced morphological changes, resulting in higher IC_50_ values for the image-based metric, as was the case with cytarabine in HCT116 and PANC-1 cell lines. The multiparametric metric, by integrating multiple morphological descriptors across the entire spheroid, remains sensitive to subtle phenotypic alterations and therefore allows IC_50_ estimation in situations when classical sigmoidal dose–response curves cannot be reliably fitted, as was the case with the LoVo cell line and the 5-FU compound.

Accordingly, the multiparametric metric is particularly informative for weak or gradual drug effects, heterogeneous spheroid morphologies, and cell lines exhibiting non-classical dose–response behavior, such as LoVo and CFPAC-1. Conversely, the proliferation-based IC_50_ values remain a robust and efficient readout when strong cytotoxicity changes together with pronounced loss of metabolic activity. Considered together, divergent IC_50_ estimates should be viewed as complementary measurements that provide insights into various layers of drug response within 3D spheroid models.

### 3.4. Validation of the PCA-Based Feature Weighting Algorithm

**Figure 7:**
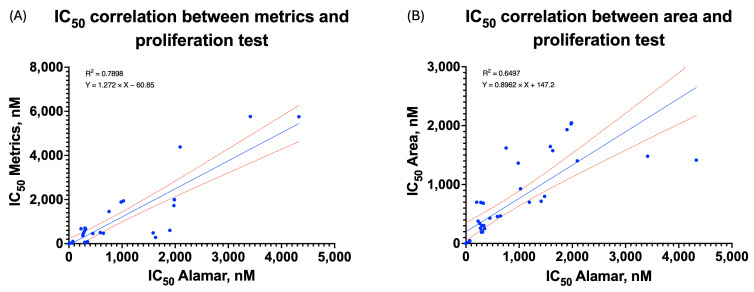
Correlation between IC_50_ of the proliferation assay and the metric (**A**) (r = 0.89, ρ = 0.91, *p* < 0.001) or between IC_50_ of the proliferation assay and the area (**B**) (r = 0.81, ρ = 0.94, *p* < 0.001).

The main aim of the conducted verification was to confirm the validity of our PCA-based approach for feature weighting by comparing the algorithm performance against the traditional single-parameter assessment and proliferation assays. The weighted metric showed a strong correlation with our proliferation assay IC_50_ values in the case of a majority of compounds and cell lines. The Pearson correlation coefficient was 0.89, *p* < 0.001 (Figure 7A), which showed a stronger correlation than the correlation between area only and the proliferation assay (r = 0.81, *p* < 0.001) (Figure 7B), except in cases when proliferation assays could not provide reliable curves. With so-called “problematic” cell lines such as LoVo and CFPAC-1, interpretable dose–response curves were obtained using our approach where traditional methods failed, therefore underscoring the utility of this strategy in analytically challenging conditions.

To address a possible non-normal distribution of IC_50_ estimates, Spearman’s rank correlation test was further applied to confirm the merits of the proposed measure (Table S3). The multiparametric measure was highly monotonically associated with IC_50_ values derived from proliferation (ρ = 0.91, *p* < 0.001). Even though IC_50_ values for the spheroid area were more highly rank-related (ρ = 0.94), their association was much weaker on a linear scale, thereby indicating that the area alone is limited for modeling dose responses.

To test the robustness of the weights obtained by PCA, the value of the IC_50_ measurement was also calculated using cumulative variance thresholds of 80% and 95%. The resulting IC_50_ values showed consistently strong Pearson and Spearman correlations with proliferation assay IC_50_ values, suggesting that the performance of the measurement was not critically affected by the choice of the cumulative variance value.

Analysis of the feature importance showed that different morphological parameters contributed almost equally to the final metric across the cell lines (Table S4). Nevertheless, area (0.13–0.16), compactness (0.13–0.15) and form factor (0.14–0.15) consistently received higher weights across all the cell lines, while parameters such as perimeter (0.11–0.14), solidity (0.11–0.15) and median radius (0.11–0.155) showed greater variability in their contributions, reflecting cell line-specific morphological responses to treatment. This differential weighting pattern supports our hypothesis that multiparametric analysis captures more comprehensive information than any single parameter alone.

It should be noted that the morphological parameters composed by the PCA-weighted metric reflect distinct and complementary features of spheroid biology and structural integrity. Spheroid area and equivalent radius-derived metrics (as Radius or Perimeter) are consequently principally modified by growth inhibition, shrinkage, or partial fragmentation with diameter increasing following cytostatic treatment [72,74,75]. Form factor and compactness are shape-describing parameters sensitive to departures from circularity and signify surface irregularity, loss of cohesion, or asymmetric growth [39,42,52]. In a somewhat related way, solidity conveys information on boundary roughness and the development of internal voids that accompany early disintegration steps, potentially preceding overt spheroid breakdown [39,76,77,78]. Texture-related features such as granularity provide information on the internal structural heterogeneity and often correlate with necrotic core formation or drug-induced disruption of spheroid architecture [79,80].

The relative importance of individual morphological parameters in PCA-weighted composite scoring varied among cell lines, representing cell line-specific intrinsic differences in spheroid morphology and responsiveness. In compact and regularly shaped spheroids, such as HCT16 and PANC-1, parameters representing size/shape information (area, median radius, form factor) were most important in the composite metric, which translated a treatment response in these models mainly into a reduced size and a morphologically altered form. In LoVo spheroids, a cell line with a more disordered heterogeneous morphology, a balanced representation of size, shape, and texture parameters in PCA composite analysis reflected a treatment response in both dimensions of size and a disordered/heterogeneous interior. Furthermore, in contrast to other cell lines, in CFPAC-1 spheroids, a higher relative importance of texture/boundary parameters in PCA composite analysis emerged in comparison with size parameters, indicating for this cell line a mainly cytostatic treatment mode of action with a focus on intracellular organization/surface integrity rather than uniform shrinkage. Together, these observations highlight that the PCA-based weighting scheme adapts to cell line–specific morphological response patterns, enabling sensitive phenotypic quantification across structurally diverse 3D spheroid models without imposing predefined feature priorities.

In this paper, an image-processing algorithm for optical images of three-dimensional cell cultures was proposed and then validated against standard proliferation assays. This algorithm development has helped overcome a significant gap in existing high-throughput screening methodologies for 3D cultures, where traditional proliferation dyes often fail to provide reliable results due to limited penetration, metabolic variations, or complex spheroid architectures [81]. In contrast, our approach takes advantage of multiparametric image analysis to derive meaningful data from label-free brightfield images, both at a lower cost and with workflow advantages compared to fluorescence-based techniques, which require additional reagents and extra imaging time.

Several alternative approaches to multiparametric morphological profiling were recently introduced, including fluorescence-based cell painting methods that employ machine learning models [33,36]. These approaches allow the generation of high-dimensional phenotypic signatures and are well-suited for mechanism-of-action analyses [34,44]. Nevertheless, they are usually based on multichannel fluorescence staining, require substantial training datasets, and might be less suitable for high-throughput 3D spheroid screening analyses due to associated costs and penetration limitations.

By contrast, our method takes label-free brightfield microscopy images and relies upon the PCA process for its data-transparent and data-driven merging of morphometric parameters with a final metric. The proposed method should be seen not as an alternative approach for classification using Cell Painting or for machine learning algorithms, but as a complementary approach, optimized for robustness and for its use in heterogeneous 3D spheroids that are difficult to assess using proliferation assays or comprehensive phenotypic profiling.

Despite its cost-effectiveness and throughput advantages, brightfield imaging has limited access to intracellular and molecular-level information, for example, nuclear morphology, apoptotic indicators, and metabolic activities [9]. These metrics may provide biological information on complex 3D spheroids that cannot be fully deduced from exterior morphology alone [18]. However, due to its label-free nature and low assay perturbation, brightfield-based analysis is still attractive for high-throughput and industrial screening [42]. Crucially, the PCA-based multiparametric framework presented here is easily expandable and may comprise inexpensive fluorescent readouts such as nuclear or live/dead staining in subsequent studies, enabling hybrid imaging approaches balancing assay scalability and biological depth.

The mathematical foundation of our approach—using PCA for feature weighting—represents a new application of dimensionality reduction techniques to spheroid analysis. Although PCA has been extensively applied to the analysis of a wide range of biomedical data for visualization and pattern recognition, to our knowledge, its application in 3D culture analysis for weighted feature integration is unprecedented [43,70,71]. This allows for objective, data-driven determination of parameter importance, eliminating subjectivity related to manual weight assignment and accounting for nonlinear relationships between the different morphological features and biological responses.

The IC_50_ values obtained for the parameters presented in this work are relatively similar compared to the results from the commonly used proliferation assay. In difficult cases, at least values that were not possible to determine using the proliferation assay could be obtained due to cell line characteristics and/or growth conditions, as shown for the LoVo cell line with 5-FU or Etoposide compounds. This may include factors such as spheroid structure, cell line proliferation properties, and individual cell sensitivity to specific compounds, among others.

The Python script developed for the multiparametric analysis of extracted metrics is provided in the Supplementary Materials, which can be further optimized and used in a high-throughput screening approach. Future validations may include high-throughput screenings of candidate therapeutic molecules and an assessment of their potential for further selection in preclinical and clinical trials.

## 4. Conclusions

During the study, we identified cell lines, mainly LoVo and CFPAC-1, for which the proliferation activity assessment, by means of Alamar Blue assay, was not reliable due to the high morphological heterogeneity of the spheroids. For an exact assessment of compound effects on these models, we developed an image analysis pipeline that includes the extraction of morphological features of spheroids using CellProfiler, with their further re-interpretation in terms of size/structure changes.

Since this approach only partly compensated for the shortcomings of metabolomic analysis and did not provide sufficient information, an additional strategy of integration of morphological parameters was developed using a Python script. Using principal component analysis, a metric was created as one comparative indicator that allowed the quantitative assessment of the effect of compounds in cases when the fluorescent signal is distorted.

This methodology has been validated with a wide variety of pancreatic and colorectal cell lines (PANC-1, CFPAC-1, HCT116, LoVo) under standard cytostatic compounds, thus extending its applicability and robustness. Application of the metric unveiled effects of 5-fluorouracil and etoposide on the LoVo cell line and showed high concordance with IC_50_ analysis data for models without metabolic readout issues (r = 0.896, ρ = 0.91, *p* < 0.001), confirming its suitability for high-throughput screening.

It brings several advantages compared to the available techniques: label-free analysis without the use of expensive fluorescent reagents, compatibility with standard brightfield imaging setups, and the extraction of multidimensional data from a single set of images. For those reasons, the approach is of particular value in industrial drug discovery pipelines, where cost-effectiveness combined with throughput represents critical features.

The Python code developed herein for multiparametric analysis is provided in Supplementary Materials to facilitate its adoption by other researchers and its potential integration into workflows already using high-throughput screening. Future directions include the extension of the present approach to more complex 3D models that take into consideration further elements of the tumor microenvironment and adaptation of the methodology for patient-derived organoids to support personalized medicine applications.

## Figures and Tables

**Table 1:** Selected conditions used for further screening analysis.

Cell Line	Pathology	Seeding Density (Cells/Well)
HCT116	Colorectal Cancer	500
LoVo	Colorectal Cancer	100
PANC-1	Pancreatic Cancer	1000
CFPAC-1	Pancreatic Cancer	1000

**Table 2:** Comparison of IC_50_ values derived from proliferation assays, area measurements, and the multiparametric metric across the studied cell lines. Values are presented as mean ± SD calculated from n = 3 independent experiments.

Cell Line	Compound	IC_50_ ± SD, nM (Proliferative)	IC_50_ ± SD, nM (Metric)	Relation Metric: Proliferative	IC_50_ ± SD, nM (Area)	Relation Area: Proliferative
HCT116	Paclitaxel	0.284 ± 0.05	0.33 ± 0.03	1.17	0.2686 ± 0.05	0.95
	5-FU	921 ± 146	1762 ± 264	1.91	1304 ± 350	1.42
	Cytarabine	303 ± 11.5	644 ± 72	2.13	198.5 ± 10.7	0.66
	Niraparib	3279 ± 1124	5300 ± 794	1.62	1432 ± 42	0.44
	Etoposide	>2000	>2000	n/a ^2^	>2000	n/a ^2^
	Oxaliplatin	>10,000	>2000	n/a ^2^	>2000	n/a ^2^
LoVo	Paclitaxel	n/a ^1^	>8	n/a ^2^	12.9 ± 13.2	n/a ^2^
	5-FU	n/a ^1^	0.0014 ± 0.0002	n/a ^2^	688 ± 253	n/a ^2^
	Cytarabine	14.2 ± 0.4	15.2 ± 9.2	1.07	14.3 ± 0.3	1.01
	Niraparib	270 ± 63	>400	>1.5	693 ± 10	2.57
	Etoposide	n/a ^1^	>80	n/a ^2^	283 ± 14	n/a ^2^
	Oxaliplatin	n/a ^1^	>80	n/a ^2^	>400	n/a ^2^
PANC-1	Paclitaxel	1.24 ± 0.04	1.28 ± 0.26	1.03	1.31 ± 0.60	1.06
	5-FU	1361 ± 150	>400	>0.29	738 ± 53	0.54
	Cytarabine	254 ± 23.5	498 ± 160	1.96	325 ± 62	1.28
	Niraparib	563 ± 102	478 ± 22	0.85	451 ± 19	0.80
	Etoposide	1982 ± 5	1904 ± 154	0.96	2041 ± 9	1.03
	Oxaliplatin	1705 ± 170	457 ± 162	0.27	1717 ± 189	1.01
CFPAC-1	Paclitaxel	0.01 ± 0.01	0.012 ± 0.002	1.20	0.011 ± 0.001	1.10
	5-FU	328 ± 27	71.8 ± 18.3	0.22	286 ± 30	0.87
	Cytarabine	71.0 ± 3.4	66.0 ± 35.3	0.93	43.3 ± 4.2	0.61
	Niraparib	>3.2	>80	n/a ^2^	>80	n/a ^2^
	Etoposide	>2000	>2000	n/a ^2^	>400	n/a ^2^
	Oxaliplatin	>10,000	527 ± 54	>0.05	>400	n/a ^2^

n/a ^1^—IC50 values could not be determined due to high data variability and failure to fit a four-parameter curve. n/a ^2^—relation could not be determined due to a lack of a clear IC50 value.

## Data Availability

The Python scripts used for data normalization, PCA, and metric computation, as well as the CellProfiler pipeline, are provided in the Supplementary Materials of this article. Raw image data and certain experimental datasets were generated in collaboration with JSC BIOCAD and are subject to data use restrictions; these data are not publicly available but may be obtained from the corresponding authors upon reasonable request and with permission of JSC BIOCAD.

## Supplementary Materials

The following supporting information can be downloaded at https://www.mdpi.com/article/10.3390/biomedicines14010108/s1. The Supporting Information contains additional experimental data mentioned in the original text of the article: Additional Python script scheme, summary of weighting factors, summary of correlations between different thresholds in PCA, and explanation of the formula for extracting metadata in CellProfiler. Figure S1: Summary of the Python script analysis algorithm; Table S1: Summary of modules used in CellProfiler pipeline; Table S2: Explanation of the formula for extracting metadata using regular expressions in CellProfiler; Table S3: Summary of correlations between different thresholds in PCA for the final metric, *p* < 0.001; Table S4: Summary of weighting factors of various morphological parameters used to determine the final metric.
