# Supplementary material for: Visualization, Data Extraction, and Multiparametric Analysis of 3D Pancreatic and Colorectal Cancer Cell Lines for High-Throughput Screening
Source: Biomedicines. 2026 Jan 6;14(1):108. doi: 10.3390/biomedicines14010108 (PMC12839131; doi:10.3390/biomedicines14010108)
Supplement: Supplementary file 1 [file biomedicines-14-00108-s001.zip › biomedicines-4013349-supplementary.pdf]

Supporting Information

Visualization, Data Extraction, and Multiparametric Analysis of 3D Pancreatic and Colorectal Cancer cell lines for high-throughput screening

Mikhail Trofimov [1, 2]*, Ilya Bulatov [1], Ilona Donskaia [1], Aleksandr Efremov [1], Ekaterina Litau [1], Velemir Lavrinenko [1], Vladimir Popov [1], Varvara Petrova [1], Anton Bukatin [2] and Stanislav Tyazhelnikov [1*]

[1] JSC BIOCAD; 198515, Saint Petersburg, Intracity Municipality the Settlement of Strelna, ul. Svyazi, d. 38, str. 1, pomeshch. 89, Russian Federation;
[2] Saint Petersburg National Research Academic University of the Russian Academy of Sciences; 194021, ul. Khlopina 8/3, Saint Petersburg, Russian Federation;
* Email: mih.andr.trofimov@gmail.com;
* Email: gvelvv@gmail.com

Table of Contents

List of Figures

Figure S1. Summary of the Python script analysis algorithm 3

Table S1. Summary of modules used in CellProfiler pipeline.......... 3

Table S2. Explanation of the formula for extracting metadata using regular expressions in CellProfiler. 7

Table S3. Summary of correlations between different thresholds in PCA for the final metric, p<0.001 8

Table S4. Summary of weighting factors of various morphological parameters used to determine the final metric.......... 8

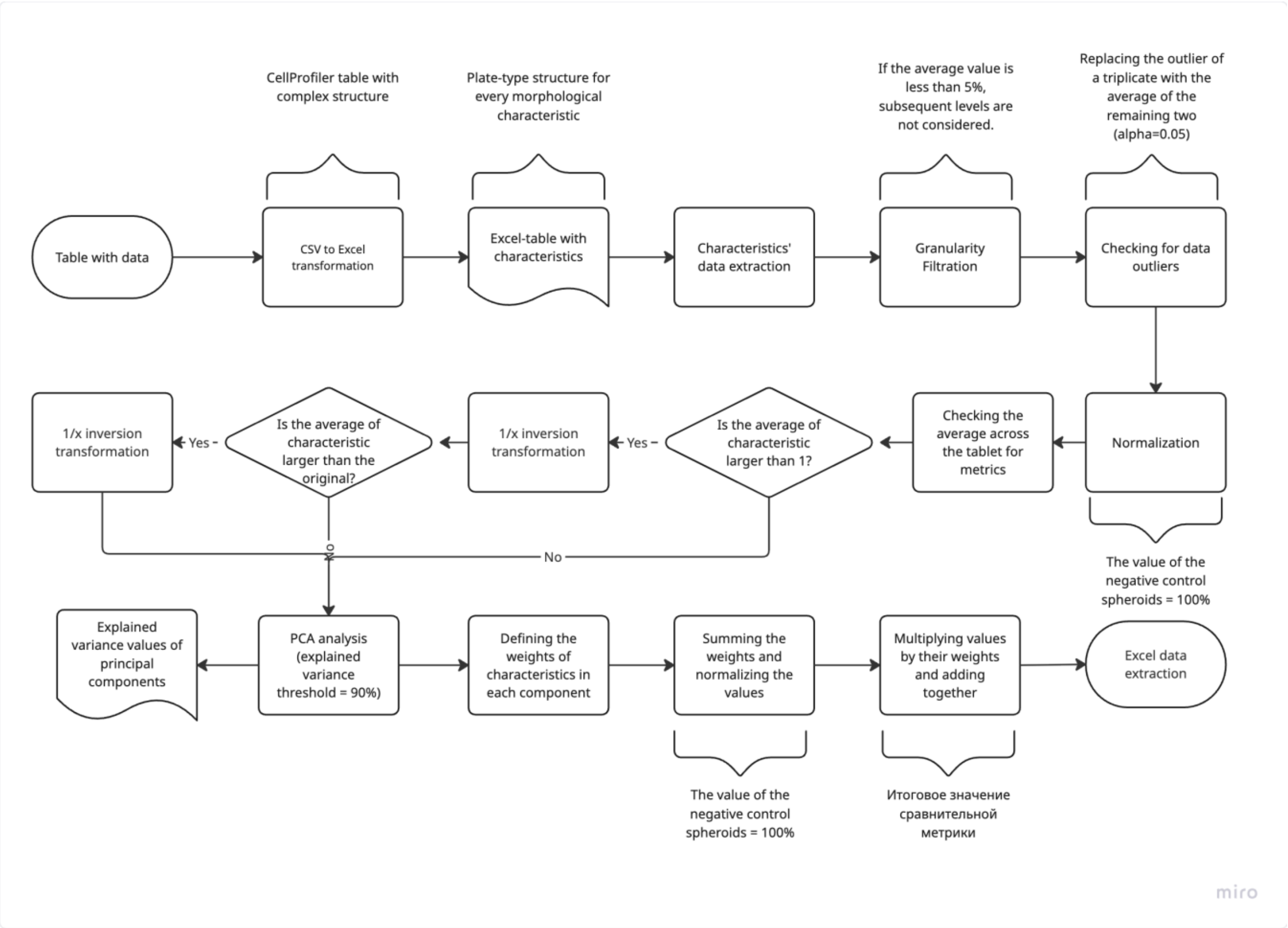

Figure S1. Summary of the Python script analysis algorithm

Table S1. Summary of modules used in CellProfiler pipeline.

Module	Function	Usage purpose	Output
		Image Preparation	
Rescale Intensity	Adjusts brightness relative to the entire image set	Normalize imaging conditions over time and staining intensity	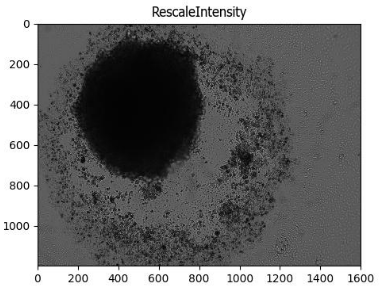

Image math	Inverts the image and enhances contrast between bright and dark pixels	Convert image to a purely black-and-white format and remove noise from potential fluctuations	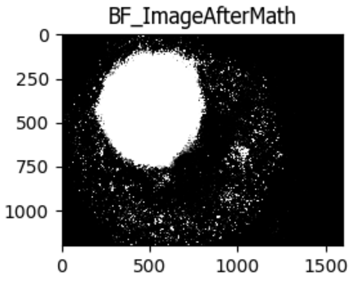
Primary Image Analysis			
Identify primary objects	Filters objects and retains those within 1-10,000 pixels after thresholding	Identify objects (pixel areas forming spheroids)	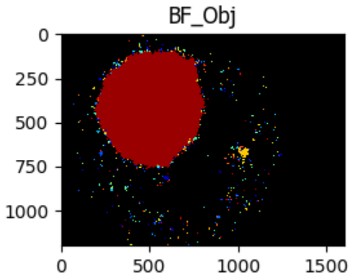
Measure object size shape	Determines the sizes of all identified objects	Assess object sizes for further filtering	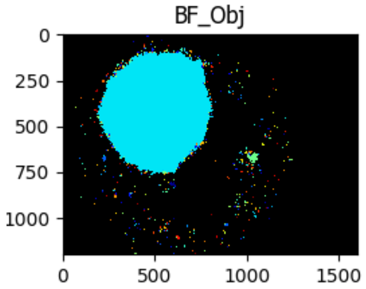
Filter objects	Removes objects smaller than 800 pixels*	Eliminate non-spheroid objects and separate them for individual analysis	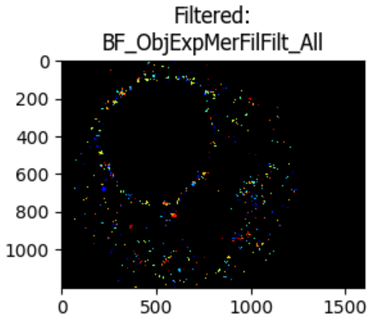
Filter objects	Collects objects larger than 800 pixels*	Group large objects separately for further analysis	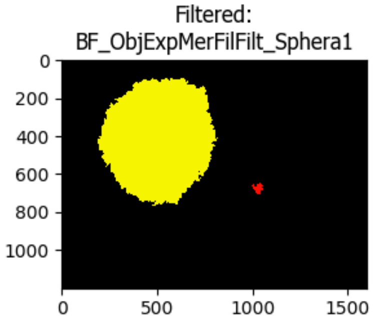

Split or Merge Objects	Merges detected objects within 1000 pixels	Unify large elements or spheroids with protrusions into a single entity (alternative to Alsehli et al. [38])	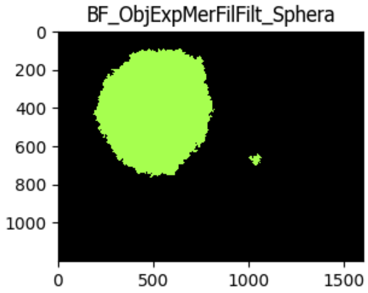
Filter objects	Collects objects smaller than 800 pixels*	Group single cells and smaller objects (if further analysis needed)	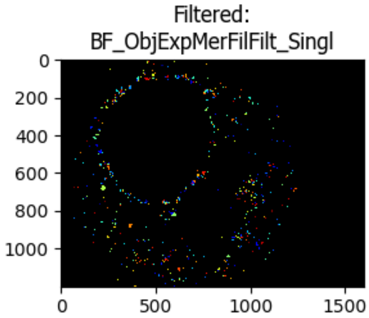
Feature Extraction			
Measure object size shape	Measures morphological features of spheroids	Extract characteristics such as perimeter and form factor	
Measure Granularity	Assesses granularity of images via iterative brightness reduction for specified radii	Evaluate image homogeneity and approximate structure size	
Measure Image Area Occupied	Measures image area characteristics in pixels	Assess spheroid size parameters	
Export to spreadsheet	Transfers all obtained data to a sheet for analysis	Prepare data for further processing	
Image and Data Extraction			
Convert objects to image	Converts identified spheroids into images	Obtain spheroid images for verification	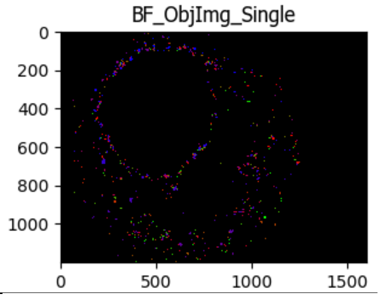
Convert objects to image	Converts identified single cells surrounding spheroids into images	Extract surrounding structures for additional analysis	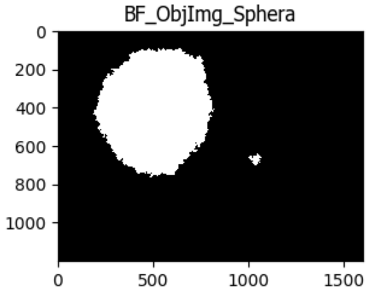

Image math	Calculates absolute pixel intensity differences between images (spheroid, single cells, background-adjusted image), creating a composite image	Generate a summary image to evaluate pipeline accuracy	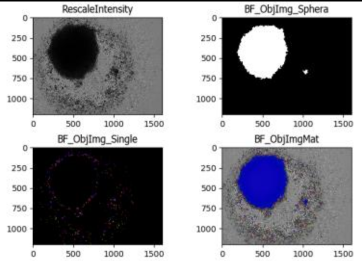
Save images	Exports processed images	Store images for record-keeping and further analysis	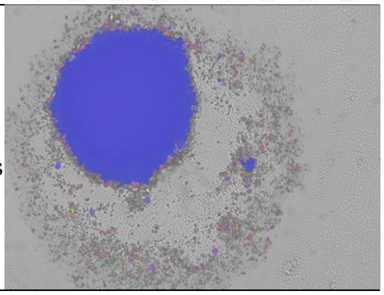

* - Object size threshold depends on the cell line.

Table S2. Explanation of the formula for extracting metadata using regular expressions in CellProfiler.

File name:
Throughout this study, all image files were named as "yyyy-mm-dd_CellLine_Exp_Name_PlateN_4X_BF_B02f00030002"

Formula:
^(?P<Date>[0-9-I]{1,99})_(?P<Cell_line>[A-Za-z0-9]{1,99})_(?P<Exp_name>[\w]{1,99})(?P<Plate>[A-Za-z0-9-|])_4X_(?P<Channel>[A-Z]{2,4})_(?P<Well>[A-P][0-9]{2}), where

Metadata name	Formula name	Explanation
yyyy-mm-dd	Date	The date of the experiment
CellLine	Cell_line	Cell line name
Exp_Name	Exp_name	Short name of the experiment
PlateN	Plate	Plate number in imaging
4X	4X	Objective magnification
_BF	Channel	Bright-field (BF) imaging indicator
B02	Well	Plate well's position

Table S3. Summary of correlations between different thresholds in PCA for the final metric, p<0.001

Type of analysis	Pearson r against 90% threshold metric	Spearman ρ against 90% threshold metric	Pearson r against alamar blue test	Spearman ρ against alamar blue test
80% Variance Threshold	0,976	0,985	0,855	0,903
90% Variance Threshold	–	–	0,89	0,913
95% Variance Threshold	0,989	0,988	0,896	0,907
Square	–	–	0,806	0,938

Table S4. Summary of weighting factors of various morphological parameters used to determine the final metric

Cell line	Area	Compactness	Form Factor	Median Radius	Perimeter	Solidity	Granularity	Granularity 2nd level
HCT116	0,151	0,146	0,154	0,154	0,112	0,140	0,143	
LoVo	0,160	0,150	0,150	0,139	0,134	0,126	0,141	
PANC-1	0,145	0,128	0,154	0,155	0,116	0,149	0,153	
CFPAC-1	0,128	0,131	0,138	0,112	0,140	0,112	0,119	0,119
